# Prediction of Depression in Women With Metabolic Dysfunction-Associated Fatty Liver Disease Using Routine Blood Tests: A Five-Year Longitudinal Analysis From the UK Biobank

**DOI:** 10.31083/AP46337

**Published:** 2026-06-17

**Authors:** Junrong Li, Taolong Zhou, Zhongwen Feng, Xiaobing Zhai, Xiaoliang Li, Tao Luo, Henry Hoi Yee Tong, Kefeng Li, Mingxing Huang

**Affiliations:** ^1^Centre for Artificial Intelligence Driven Drug Discovery, Faculty of Applied Sciences, Macao Polytechnic University, 999078 Macau, China; ^2^Department of Infectious Diseases, Zhuhai Third People’s Hospital, 519099 Zhuhai, Guangdong, China

**Keywords:** non-alcoholic fatty liver disease, depression, algorithm, female, machine learning, risk assessment

## Abstract

**Background:**

Metabolic dysfunction-associated fatty liver disease (MAFLD) affects 38.9% of the global adult population and is associated with increased mortality when it co-occurs with depression. Women exhibit a 1.5– to 3–fold higher prevalence of depression, with postmenopausal hormonal imbalances amplifying susceptibility. This underscores the urgent need for sex-specific predictive models. The aim of this study was to develop a lightweight, high-accuracy model to identify key predictors of depression risk in female MAFLD patients using a five-year longitudinal UK Biobank cohort.

**Methods:**

We analyzed routine blood biomarkers (hematology and metabolites), lifestyle factors, and reproductive history from female participants with MAFLD identified within the UK Biobank cohort. Logistic regression was adjusted for socioeconomic, lifestyle, multimorbidity, and medication use, and was employed to evaluate sex-specific factors. Feature selection employed a two-stage approach to ensure balanced covariate distributions between cases and controls: Random Forest-based stratified bootstrap resampling (1000 iterations) instead of traditional random sampling, followed by recursive feature elimination. Five models—Light Gradient Boosting Machine (LightGBM), Extreme Gradient Boosting (XGBoost), Random Forest, Feature Tokenizer Transformer (FT-Transformer), and Gated Adaptive Network for Deep Automated Learning of Features (GANDALF)—were assessed via 10–fold cross-validation. SHapley Additive exPlanations (SHAP) and restricted cubic splines (RCS) elucidated feature importance and nonlinear effects.

**Results:**

A total of 39,430 female MAFLD patients were included in the final analysis, among whom 611 (1.55%) developed incident depression over the five-year follow-up. Adjusted logistic regression identified younger age at first live birth (<20 years) and early menopause (<35 years) as significant risk factors for depression. Among the evaluated models, GANDALF demonstrated superior performance (area under the receiver operating characteristic curve [ROC-AUC] = 0.96 ± 0.03, Matthews’ correlation coefficient [MCC] = 0.830 ± 0.068), significantly outperforming conventional machine learning approaches (MCC range: 0.720 to 0.760) and exhibiting better calibration (Brier score: 0.066 vs. 0.093–0.115). SHAP analysis identified red blood cell count, Townsend deprivation index, and neutrophil count as the most influential predictors among the 17-feature panel. RCS analyses revealed nonlinear protective effects of moderate physical activity and higher red blood cell counts, contrasted by adverse effects from elevated white blood cell counts, overweight (body mass index [BMI] >25 kg/m^2^) and obesity (body mass index [BMI] >30 kg/m^2^), and early reproductive milestones. Additionally, our feature set showed enhanced predictive validity compared to established biomarker panels from prior studies (ROC-AUC 0.96 vs. 0.886–0.940).

**Conclusions:**

This study introduces a highly accurate, lightweight predictive model tailored for female MAFLD patients, leveraging 17 key features to improve the prediction of depression risk. By enabling personalized risk assessment and targeted interventions, our model offers a transformative approach to improve mental health outcomes and care quality in this vulnerable population.

## Main Points

1. Adjusted analyses revealed that early age at first live birth (<20 years) and premature menopause (<35 years) are independent risk factors for depression. These associations persisted even after controlling for lifestyle factors, metabolic comorbidities, and medication use.

2. Utilizing a 17-feature panel identified through robust feature selection, the Gated Adaptive Network for Deep Automated Learning of Features (GANDALF) model demonstrated superior performance over conventional machine learning approaches in predicting depression risk, achieving an area under the curve (AUC) of 0.96 and a Brier score of 0.066.

3. The developed model generates personalized depression risk scores for patients with MAFLD, thereby facilitating early clinical intervention and optimizing prognostic management.

## 1. Introduction

Metabolic dysfunction-associated fatty liver disease (MAFLD) is widely recognized as the hepatic manifestation of metabolic syndrome, presenting a growing challenge to global health. The prevalence of fatty liver disease has increased significantly from 18.2% in 1990 to 38.9% in 2020, with a projected rise to 55.7% by 2040 [1]. As part of this rising trend, epidemiological evidence has highlighted a pronounced gender dimorphism. In particular, postmenopausal women exhibit a progressive increase in the incidence of MAFLD, peaking between the ages of 60 and 69 years [2]. This age-related vulnerability is particularly concerning given the potential of MAFLD to progress to severe liver damage and its established associations with systemic comorbidities, including type 2 diabetes mellitus, cardiovascular disease, and psychiatric disorders [3]. Notably, women aged ≥50 years are 1.2–fold more likely to develop non-alcoholic steatohepatitis (NASH) and experience accelerated progression to advanced liver fibrosis compared to age-matched men [4, 5].

Converging evidence reveals a bidirectional pathogenic relationship between MAFLD and depression. A large cross-sectional analysis of 10,484 subjects found a significantly higher prevalence of depression and depression-related functional impairment among individuals with MAFLD than in those without [6]. This association confers severe clinical implications, since depression comorbidity in MAFLD patients doubles cardiovascular event rates, compromises treatment adherence, and increases all-cause mortality [7]. Longitudinal data further substantiate these findings, with an 81% higher risk of cognitive decline observed in MAFLD individuals in a study of 845 participants [4].

Importantly, women exhibit a 1.5– to 3–fold higher prevalence of depression than men, a disparity amplified by postmenopausal status. This increased susceptibility is rooted in complex biological dimorphisms involving immune, metabolic, and neuroendocrine systems. Specifically, elevated levels of inflammatory biomarkers, such as C-reactive protein (CRP) and IL-6, have been positively associated with depressive symptoms in women, but not consistently in men [8]. Furthermore, the interaction between the hypothalamic-pituitary-adrenal (HPA) axis and fluctuating sex hormone levels (e.g., estrogen withdrawal) sensitizes women to stress-induced neuroinflammation [9]. In the context of MAFLD, these vulnerabilities are exacerbated by the gut-liver-brain axis. Metabolic dysfunction and visceral adiposity are regulated by gonadal hormones and can synergize with gut-derived endotoxins to amplify systemic inflammation [10]. Consequently, the translocation of inflammatory mediators compromises the blood-brain barrier, linking liver pathology directly to mood disorders. These interconnected biological pathways highlight the urgent need for sex-specific predictive models to assess depression risk specifically in women with MAFLD, enabling timely interventions to mitigate multifaceted health risks.

Despite growing recognition of this comorbidity, the underlying mechanisms and risk factors remain poorly characterized, particularly those operating through sex-specific biological pathways. While predictive models for depression in women already exist, they rely predominantly on subjective behavioral assessments, lack objective biological biomarkers, and frequently overlook critical determinants such as hormonal influences and reproductive life stages [11]. Based on these biological underpinnings, we hypothesized that the incorporation of sex-specific reproductive and biochemical features would significantly improve the prediction of depression in women with MAFLD.

To address the gender issue, we leveraged a large-scale, five-year longitudinal cohort from the UK Biobank (N = 611 cases; N = 38,819 controls) to construct a high-accuracy, sex-specific predictive model. We integrated various multidimensional features, including routine blood biomarkers (hematology and metabolites), lifestyle factors, and reproductive history, to develop a ‘lightweight’ model that relies on a minimal set of robust, cost-effective, and easily accessible risk factors. Unlike cross-sectional analyses, this longitudinal design established a clear temporal sequence, confirming that metabolic dysfunction precedes depression and thereby strengthening the evidence for these biomarkers. Ultimately, this individualized risk scoring system aims to advance precision mental health care by providing a practical clinical tool for early detection and targeted intervention.

## 2. Materials and Methods

### 2.1 Data Source

This longitudinal cohort study utilized data from the UK Biobank (https://www.ukbiobank.ac.uk/), a large-scale, population-based prospective study that includes 502,536 participants aged 39–70 years and recruited between 2006 and 2010 across 22 assessment centers in the United Kingdom [12]. The methodology for the UK Biobank has been published previously and is available to the public [13]. All participants provided electronic informed consent for continuous health monitoring.

### 2.2 Sex-Specific and Clinical Data Collection

To capture the unique physiological and hormonal influences on the risk of depression among women with MAFLD, we incorporated a comprehensive panel of sex-specific variables derived from the UK Biobank touchscreen questionnaire (**Supplemental Text 1**).

The reproductive history metrics encompassed parity (total number of live births; UKB field 2734), age at first live birth (UKB field 2754), age at last live birth (UKB field 2764), and number of spontaneous miscarriages (UKB field 3839). These metrics collectively reflect the cumulative exposure to reproductive hormone fluctuations and pregnancy-related metabolic adaptations. Menopausal transitions were characterized by age at menopause (UKB field 3851) and age at hysterectomy (UKB field 2824), while age at menarche (UKB field 2714) and length of menstrual cycle (UKB field 3710) provided indices of pubertal maturation and cyclical hormonal regulation. Additionally, we incorporated data on age at initiation of hormone-replacement therapy (hormone-replacement therapy [HRT]; UKB field 3536), use of the oral contraceptive pill (UKB field 2794), and age at bilateral oophorectomy (UKB field 3882) to evaluate the impact of exogenous hormone exposure and surgical menopause on the susceptibility to depression.

Lastly, we examined 30 biochemical markers (UKB category 17518) and 28 routine blood cell counts (UKB Category 100081) that provide a detailed overview of the metabolic and liver health of participants (**Supplementary Table 1**).

### 2.3 Selection of Covariates

The association between MAFLD and depression was rigorously assessed by adjusting for a comprehensive array of covariates across multiple domains. Demographic variables included age, annual household income (<£18,000, £18,000–51,999, £52,000–100,000, and >£100,000; based on an exchange rate of £1 ≈ $1.84 USD), education level (low, moderate, or high), ethnicity (British, Irish, or other), and household size. Anthropometric indicators of general and central obesity were included, specifically the body mass index (BMI) and waist circumference. We also incorporated various lifestyle factors known to influence metabolic and mental health, including smoking status and alcohol consumption (never, previous, or current), dietary habits (vegetable, fruit, and meat intake), physical activity (quantified as total Summed MET (min) [MET]-minutes), and sleep duration. Furthermore, we accounted for major comorbidities, including cancer, hypertension, and diabetes. Finally, to mitigate confounding by pharmacotherapy, we controlled for the use of common medications recorded in the UK Biobank, specifically antidepressants (e.g., sertraline, citalopram, escitalopram, fluoxetine, venlafaxine, mirtazapine) and statins (e.g., simvastatin, atorvastatin, pravastatin, fluvastatin, rosuvastatin). We included antidepressant use as a covariate rather than an exclusion criterion to rigorously control for medication-induced metabolic confounding while maintaining robust statistical power.

### 2.4 Estimation of Missing Values

To ensure the quality and reliability of the dataset, variables with >20% missing values were excluded, and participants with >50% missing data were removed from the analysis. For the remaining data, missing values were imputed using Bayesian Principal Component Analysis (BPCA) [14], a robust and statistically sound approach to handling incomplete data. Subsequently, the dataset was partitioned into training and test sets. Continuous variables were then log-transformed and auto-scaled, with transformation parameters derived exclusively from the training data to strictly prevent data leakage, before being used in the machine learning models [15].

### 2.5 Analysis of Sex-Specific Factors 

The association between individual sex-specific factors and depression was initially assessed using univariate logistic regression. Subsequently, a hierarchical covariate adjustment process was implemented in three stages: (1) adjustment for sociodemographic variables, including age, income, education, BMI, and race; (2) further adjustment for lifestyle-related factors, such as physical activity, smoking status, alcohol consumption, and sleep duration; and (3) further control for multimorbidity (hypertension, diabetes, cancer) and medication use (antidepressants, statins). Statistical significance was determined at a threshold of *p* < 0.05. Factors demonstrating significant associations with depression in this multi-stage analysis were subsequently selected for incorporation into the final predictive model.

### 2.6 Candidate Features

To complement the sex-specific predictors, we also conducted a separate feature selection process on a candidate pool of biochemical markers, routine blood cell count parameters, and lifestyle indicators. This selection was performed in two steps: preliminary screening, followed by final subset determination. During the preliminary screening stage, we implemented a stratified bootstrap sampling strategy designed to simultaneously address class imbalance and potential confounding. Specifically, across 1000 iterations, sampling was stratified to strictly align the distributions of age and BMI between the case and control groups, maintaining a fixed 1:3 ratio. In each iteration, a random forest model ranked features based on their importance [16]. The top 50 features in each sampling were retained. At the conclusion of 1000 iterations, features were ranked by their frequency of selection. The top 20% of features, 22 in all, were shortlisted as candidates for further analysis. In the second stage, Recursive Feature Elimination (RFE) was employed to establish a hierarchy of feature importance. Subsequently, an incremental feature selection strategy was adopted, sequentially adding predictors according to their rank. To ensure clinical practicability, the search space was restricted to the top 15 features. Furthermore, Spearman rank correlation-based hierarchical clustering was applied to address potential multicollinearity among the top candidates [17]. A correlation coefficient of >0.8 between features within a cluster was considered indicative of collinearity. The final feature subset was determined by identifying the point of the highest AUC within this predefined constraint.

### 2.7 Model Construction

The performance of three machine learning algorithms (XGBoost, LightGBM, Random Forest) and two deep learning architectures (Gated Adaptive Network for Deep Automated Learning of Features [GANDALF] [18], FT-Transformer [19]) were evaluated on the selected optimal feature subset. To address the class imbalance present in the dataset, the Nearmiss undersampling technique was applied to ensure balanced training data [20]. For each model, the experiment was repeated 10 times with random data shuffling. In each run, 20% of the data was held for the independent test set, while the remaining 80% was used for training via 10–fold cross-validation. The optimization of hyperparameters was performed using GridSearchCV to identify the best parameter configurations and reduce the risk of overfitting [21]. To ensure reproducibility of the results, a fixed random seed (set to 42) was applied across all data splitting, undersampling, and model training processes.

To thoroughly evaluate model performance, a range of standard metrics was used, including accuracy, area under the receiver operating characteristic curve (ROC-AUC), F1-score, recall, Matthews’ correlation coefficient (MCC), sensitivity, and specificity. Additionally, the Brier score was calculated to assess calibration accuracy by quantifying the squared differences between predicted probabilities and observed outcomes [22]. We also computed the logarithmic loss (Log-Loss) to measure the divergence between true labels and predicted probabilities, thus providing insights into predictive uncertainty [23]. Among these metrics, MCC was prioritized as the primary evaluation criterion for the test set due to its balanced consideration of both true/false positives and negatives [24].

### 2.8 Model Interpretability

To elucidate key predictors of comorbid depression in female patients with MAFLD, we employed a dual interpretability framework integrating SHapley Additive exPlanations (SHAP) values and restricted cubic spline (RCS) modeling [25]. SHAP analysis offers both global and patient-level insights by quantifying the contribution of each clinical feature to model predictions across the entire cohort and within individual cases. This approach not only indicates the relative importance of each variable, but also facilitates the development of personalized depression risk scores, giving clinicians a detailed understanding of each patient’s unique risk profile.

To further explore the relationships between continuous clinical variables and the risk of depression, we applied RCS regression utilizing the entire dataset. This technique identified critical thresholds where the likelihood of comorbidity sharply increases, providing actionable insights for risk stratification and early intervention strategies. Statistical significance of model terms was assessed using two-tailed Wald tests, with *p*  <  0.05 considered statistically significant.

### 2.9 Statistical Analyses

For descriptive statistics, categorical variables were presented as frequencies and percentages, with the chi-square test used for comparisons. Continuous variables with a normal distribution were expressed as the mean ± standard deviation, and differences were assessed using the *t*-test. For data preprocessing, missing values were imputed using the BPCA algorithm. This was implemented via the ImputeMissingVar function within the MetaboAnalyst platform (https://new.metaboanalyst.ca/MetaboAnalyst/). Model training and visualization were performed in Python (v3.11.10; Python Software Foundation, Wilmington, DE, USA) with packages from the scikit-learn library (v1.6.1; https://scikit-learn.org/) and Shap library (v0.47.2; https://github.com/shap/shap). RCS analyses were performed using the plotRCS package (v0.1.5; https://github.com/KunHuo/plotRCS) in R (https://www.r-project.org/). All statistical tests were two-sided, and a *p*-value < 0.05 was considered statistically significant.

## 3. Results 

### 3.1 Study Population

Our analysis focused on 164,371 participants diagnosed with hepatic steatosis. Given the lack of liver imaging or histological data within the UK Biobank, the Fatty Liver Index (FLI) was employed as a surrogate marker to confirm the presence of hepatic steatosis in the baseline population (2004–2011), utilizing a validated cutoff value (FLI ≥60) [26]. Participants were further classified as having MAFLD if they met the FLI-based criteria for hepatic steatosis and did not meet any of the following exclusion criteria [27]: (1) Hospital diagnosis of liver disease due to other causes (N = 2301); (2) Hospital diagnosis of malignant liver tumors (N = 163); (3) Excessive alcohol consumption (≥30 g/day for men or ≥20 g/day for women) (Data-Field: 26030) (N = 5919); (4) Pre-existing cases of depression identified through ICD-10 and self-reported health questionnaires (N = 15,644); (5) Individuals with incomplete follow-up data (N = 36,393).

**Fig. 1.**
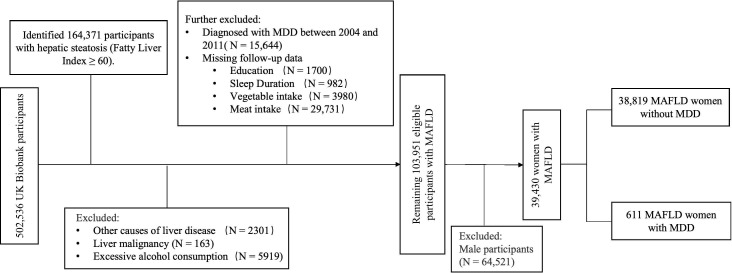
**Flowchart of the selection of eligible study population**. MDD, major depressive disorder; MAFLD, metabolic dysfunction-associated fatty liver disease.

After applying these exclusion criteria, 103,951 participants with MAFLD were included in the final analysis, of whom 39,430 were women. The mean age of these women was 58 years, and the cohort was predominantly of British ethnicity (87.3%). During the 5-year follow-up period concluding in 2015, 611 women were newly diagnosed with depression, corresponding to an incidence rate of 1.55% (Fig. 1).

### 3.2 Baseline Characteristics

Baseline analyses revealed several significant associations with the risk of depression. Women who developed depression during follow-up exhibited a significantly higher Townsend deprivation index (TDI), reflecting greater socioeconomic disadvantage. This risk pattern displayed a non-linear relationship with income, as both low- and high-income groups demonstrated a heightened susceptibility to depression compared to those with moderate income. Furthermore, depression was associated with significantly increased BMI and waist circumference among affected women. The use of prescription medications was also more common among women with depression, suggesting potential contributions from pharmacological treatments or underlying comorbidities to depressive symptoms. Additionally, the presence of comorbidities, including diabetes and cancer, was shown to substantially increase the risk of depression. Baseline characteristics and key predictors of depression at 5-year follow-up are summarized in Table 1. Detailed blood biochemistry and hematologic parameters are provided in **Supplementary Table 2**.

**Table 1.** **Baseline characteristics and 5-year follow-up comparison of MAFLD patients with and without depression**.

		Total	MAFLD without depression	MAFLD with depression	*p*.overall
		N = 39,430	N = 38,819	N = 611	
Age, median (IQR)		58 (51, 63)	58 (51, 63)	57 (50, 63)	0.361
Education, n (%)					<0.001
	Low levels of education	11,841 (30.0%)	11,660 (30.0%)	181 (29.6%)	
	Moderate levels of education	4569 (11.6%)	4512 (11.6%)	57 (9.3%)	
	High levels of education	15,876 (40.3%)	15,669 (40.4%)	207 (33.9%)	
	None of the above	7090 (18.0%)	6935 (17.9%)	155 (25.4%)	
Race/ethnicity, n (%)					0.145
	British	34,430 (87.3%)	33,881 (87.3%)	549 (89.9%)	
	Irish	986 (2.5%)	972 (2.5%)	14 (2.3%)	
	Other	4014 (10.2%)	3966 (10.2%)	48 (7.86%)	
Number in household, n (%)					0.055
	1	8263 (21.0%)	8125 (20.9%)	138 (22.6%)	
	2	18,636 (47.3%)	18,361 (47.3%)	275 (45.0%)	
	3	6160 (15.6%)	6049 (15.6%)	111 (18.2%)	
	≥4	6128 (15.5%)	6048 (15.6%)	80 (13.1%)	
	Missing	243 (0.62%)	236 (0.61%)	7 (1.15%)	
Income, n (%)					<0.001
	<18,000	9294 (23.6%)	9120 (23.5%)	174 (28.5%)	
	18,000 to 51,999	17,759 (45.0%)	17,522 (45.1%)	237 (38.8%)	
	52,000 to 100,000	6736 (17.1%)	6678 (17.2%)	58 (9.5%)	
	>100,000	5641 (14.3%)	5499 (14.2%)	142 (23.2%)	
TDI, median (IQR)		–1.87 (–3.47, 0.98)	–1.87 (–3.47, 0.97)	–1.34 (–3.31, 1.51)	0.004
Taking prescription medications, n (%)					<0.001
	No	18,010 (45.7%)	17,851 (46.0%)	159 (26.0%)	
	Yes	21,302 (54.0%)	20,852 (53.7%)	450 (73.6%)	
	Missing	118 (0.30%)	116 (0.30%)	2 (0.33%)	
Smoking status, n (%)					<0.001
	Never smoker	23,033 (58.4%)	22,723 (58.5%)	310 (50.7%)	
	Previous smoker	13,172 (33.4%)	12,967 (33.4%)	205 (33.6%)	
	Current smoker	3111 (7.9%)	3020 (7.8%)	91 (14.9%)	
	Missing	114 (0.30%)	109 (0.28%)	5 (0.82%)	
BMI, median (IQR)		31.9 (29.1, 35.3)	31.9 (29.1, 35.3)	32.9 (29.9, 36.7)	<0.001
Waist circumference, median (IQR)		97 (90, 105)	97 (90, 104)	100 (92, 107)	<0.001
Summed MET minutes/week, median (IQR)		1525 (660, 2952)	1515 (658, 2970)	2356 (872, 2356)	0.014
FLI, median (IQR)		78.1 (68.7, 88.4)	78.0 (68.7, 88.4)	81.3 (70.2, 91.2)	<0.001
Fruit intake, pieces/day, median (IQR)		4.00 (3.00, 5.00)	4.23 (2.00, 4.00)	4.32 (2.00, 4.00)	0.469
Vegetable intake, tablespoons/day, median (IQR)		6.00 (4.00, 7.00)	6.17 (3.00, 6.00)	6.01 (3.00, 6.00)	0.286
Meat intake/week, n (%)					0.771
	Never	1260 (3.2%)	1245 (3.21%)	15 (2.45%)	
	<1 time	8044 (20.4%)	7918 (20.4%)	126 (20.6%)	
	1 time	27,682 (70.2%)	27,251 (70.2%)	431 (70.5%)	
	≥2 times	2444 (6.20%)	2405 (6.20%)	39 (6.38%)	
Diastolic blood pressure, median (IQR)		84.5 (78.0, 90.0)	84.5 (78.0, 90.0)	84.5 (77.5, 89.5)	0.367
Systolic blood pressure, median (IQR)		139 (127, 150)	138 (127, 150)	138 (126, 146)	0.026
CVD, n (%)					0.083
	No	37,495 (95.1%)	36,924 (95.1%)	571 (93.5%)	
	Yes	1868 (4.70%)	1828 (4.71%)	40 (6.55%)	
	Missing	67 (0.20%)	67 (0.17%)	0 (0.00%)	
Hypertension, n (%)					0.061
	No	26,527 (67.3%)	26,138 (67.3%)	389 (63.7%)	
	Yes	12,903 (32.7%)	12,681 (32.7%)	222 (36.3%)	
Diabetes, n (%)					0.002
	No	36,311 (92.1%)	35,771 (92.1%)	540 (88.4%)	
	Yes	2994 (7.6%)	2927 (7.54%)	67 (11.0%)	
	Missing	125 (0.3%)	121 (0.31%)	4 (0.65%)	
Cancer, n (%)					<0.001
	No	35,586 (90.3%)	35,055 (90.3%)	531 (86.9%)	
	Yes	3673 (9.3%)	3603 (9.3%)	70 (11.5%)	
	Missing	171 (0.4%)	161 (0.41%)	10 (1.64%)	
Long-standing illness, disability or infirmity, n (%)					<0.001
	No	24,004 (60.9%)	23,760 (61.2%)	244 (39.9%)	
	Yes	14,483 (36.7%)	14,142 (36.4%)	341 (55.8%)	
	Missing	943 (2.40%)	917 (2.36%)	26 (4.26%)	
Grip strength (right), median (IQR)		24 (20, 29)	24 (20, 29)	22 (18, 28)	<0.001
Grip strength (left), median (IQR)		22 (18, 27)	22 (18, 27)	21 (16, 26)	<0.001
Age at menarche, median (IQR)		13 (11, 14)	13 (11, 14)	13 (11, 14)	0.226
Length of menstrual cycle, median (IQR)		26 (–1, 28)	26 (–1, 28)	26.0 (–1, 28)	0.608
Age at hysterectomy, median (IQR)		43 (38, 49)	43 (38, 49)	42 (36, 46)	0.001
Age started HRT, median (IQR)		47 (42, 50)	47 (42, 50)	45 (40, 50)	<0.001
Age at bilateral oophorectomy, median (IQR)		48 (42, 52)	48 (42, 52)	45 (41, 50)	0.019
Number of live births, median (IQR)		2 (1, 3)	2 (1, 3)	2 (1, 3)	0.427
Age at first live birth, median (IQR)		24 (21, 28)	24 (21, 28)	23.5 (20, 27)	<0.001
Age at last live birth, median (IQR)		30 (26, 33)	30 (26, 33)	29 (26, 33)	0.098
Number of spontaneous miscarriages, median (IQR)		1 (0, 1)	1 (0, 1)	1 (0, 1)	0.427
Age of primiparous women at birth of child, median (IQR)		28 (24, 33)	28 (24, 33)	28 (24, 33)	0.505
Age at menopause, median (IQR)		50 (46, 53)	50 (46, 53)	50 (43, 52)	0.001

IQR, interquartile range; TDI, Townsend deprivation index; BMI, body mass index; MET, Summed MET (min); FLI, Fatty Liver Index; CVD, cardiovascular disease; HRT, hormone-replacement therapy.

### 3.3 Sample Size Estimation 

To ensure adequate statistical power for reliable model performance, we performed a sample size calculation using the Power Analysis module in MetaboAnalyst 4.0. As shown in **Supplementary Fig. 1**, this analysis revealed a direct relationship between increasing sample size and enhanced predictive accuracy, with the recommended power threshold of 80% achieved at n = 1000 samples. Building on this empirical validation, a sample size greater than this critical threshold was selected to guarantee adequate sensitivity for the detection of biologically significant effect sizes, while also minimizing the risk of Type II errors.

### 3.4 Association of Sex-Specific Factors With Depression

**Fig. 2.**
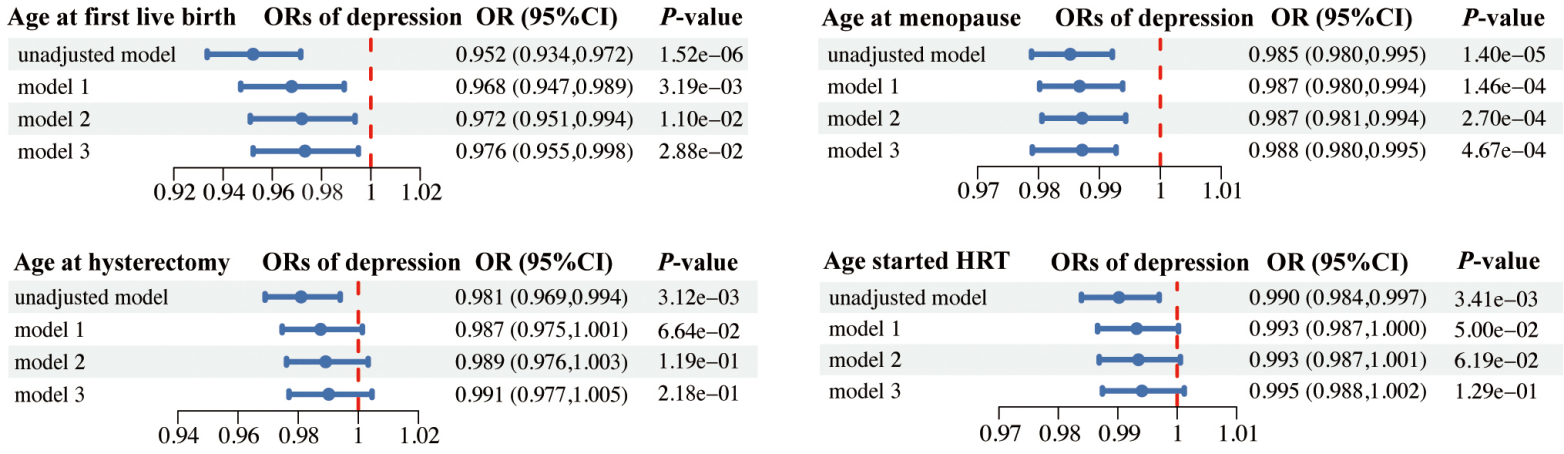
**Logistic regression models are used to assess the association between sex-specific factors and the incidence of depression**. Only the statistically significant associations are displayed herein. Complete logistic regression results for all sex-specific variables are provided in **Supplementary Table 3**. We constructed three sequentially adjusted logistic regression models: Model 1: Adjusted for core sociodemographic covariates (age, ethnicity, educational attainment, BMI, household income); Model 2: Model 1 + lifestyle factors (smoking status, frequency of alcohol consumption, sleep quality, physical activity level); Model 3: Model 2 + major comorbidities (hypertension, diabetes, cancer history) and medication use (antidepressants and statins). Abbreviations: HRT, hormone-replacement therapy; OR, odds ratio; CI, confidence interval.

As illustrated in Fig. 2 (**Supplementary Table 3**), univariate logistic regression in the unadjusted model identified several sex-specific factors that were significantly associated with depression: earlier age at first live birth (odds ratio [OR] = 0.952, 95% confidence interval [CI]: 0.934–0.972, *p* = 1.52 × 10^-6^), earlier age at menopause (OR = 0.985, 95% CI: 0.979–0.992, *p* = 1.4 × 10^-5^), earlier age at hysterectomy (OR = 0.981, 95% CI: 0.969–0.994, *p* = 3.12 × 10^-3^), and earlier initiation of HRT (OR = 0.990, 95% CI: 0.984–0.997, *p* = 3.41 × 10^-3^).

After progressive adjustment for demographic factors (model 1), lifestyle habits (model 2), and comorbidities and medication use (antidepressants and statins; model 3), the associations between depression and age at hysterectomy (OR = 0.991, 95% CI: 0.977–1.005, *p* = 0.218), as well as age at HRT initiation (OR = 0.995, 95% CI: 0.988–1.002, *p* = 0.129), were no longer statistically significant. In contrast, the relationships between depression and age at first live birth (OR = 0.976, 95% CI: 0.955–0.998, *p* = 2.88 × 10^-2^) as well as age at menopause (OR = 0.988, 95% CI: 0.980–0.995, *p* = 4.67 × 10^-4^) remained robust. Consequently, these two variables were selected for inclusion in the final predictive model for depression.

### 3.5 Feature Selection

**Fig. 3.**
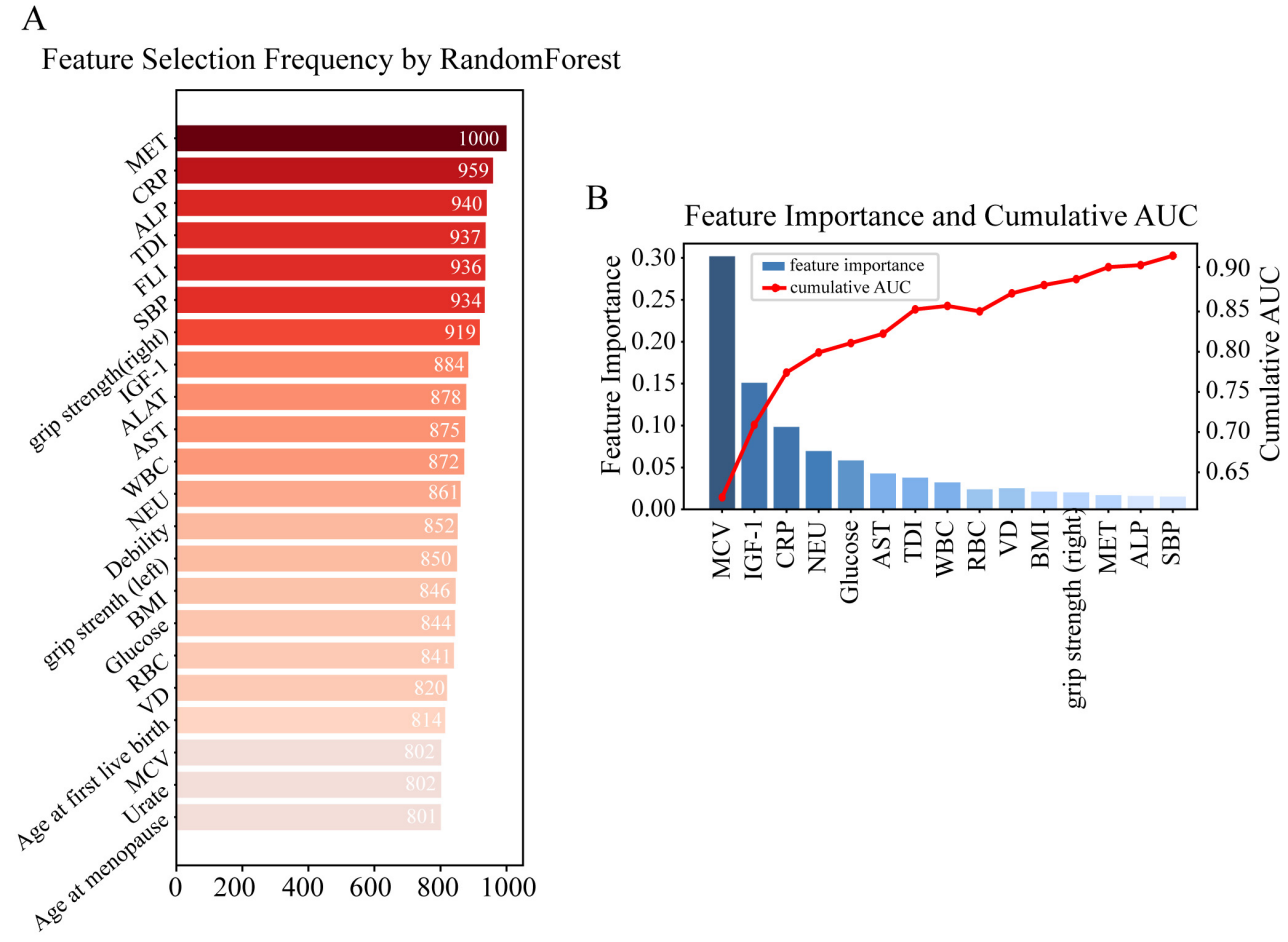
**Two-stage feature selection process for identifying robust predictors of depression in female patients with MAFLD**. (A) The top 20% of features initially selected based on their frequency ranking after 1000 bootstrap iterations. (B) The 15 predictive factors were identified through forward selection using the Random Forest model. The left y-axis represents feature importance, while the right y-axis shows the cumulative ROC-AUC. Abbreviations: CRP, C-reactive protein; ALP, Alkaline phosphatase; SBP, Systolic blood pressure; ALAT, Alanine aminotransferase; AST, Aspartate aminotransferase; WBC, White blood cell (leukocyte) count; NEU, Neutrophil count; RBC, Red blood cell count; VD, Vitamin D; MCV, Mean corpuscular volume; ROC-AUC, area under the receiver operating characteristic curve; IGF-1, insulin-like growth factor 1.

We implemented a two-stage feature selection protocol to identify robust predictors of depression in female patients with MAFLD. This leveraged biochemical markers, routine blood cell count parameters, and sex-specific factors. In the initial stage, following 1000 experimental iterations, candidate features were selected using an RF model based on their frequency of selection. This process identified 22 variables, representing 20% of the total pool, demonstrating high reproducibility across trials (Fig. 3A).

In the subsequent refinement stage, recursive feature elimination within the RF framework was employed to prioritize these features according to their contribution to model performance. This was assessed by the feature importance scores. The model’s performance, tracked by the AUC on the right axis, exhibited a sharp increase with the inclusion of the first few variables, followed by a gradual stabilization around 93% as additional variables were incorporated. Ultimately, the top 15 variables were selected as the final predictors for depression in the female MAFLD population (Fig. 3B). Validation via Spearman rank correlation-based hierarchical clustering indicated there was no multicollinearity among these final features (**Supplementary Fig. 2**).

### 3.6 GANDALF Model Selected as the Base Framework

**Fig. 4.**
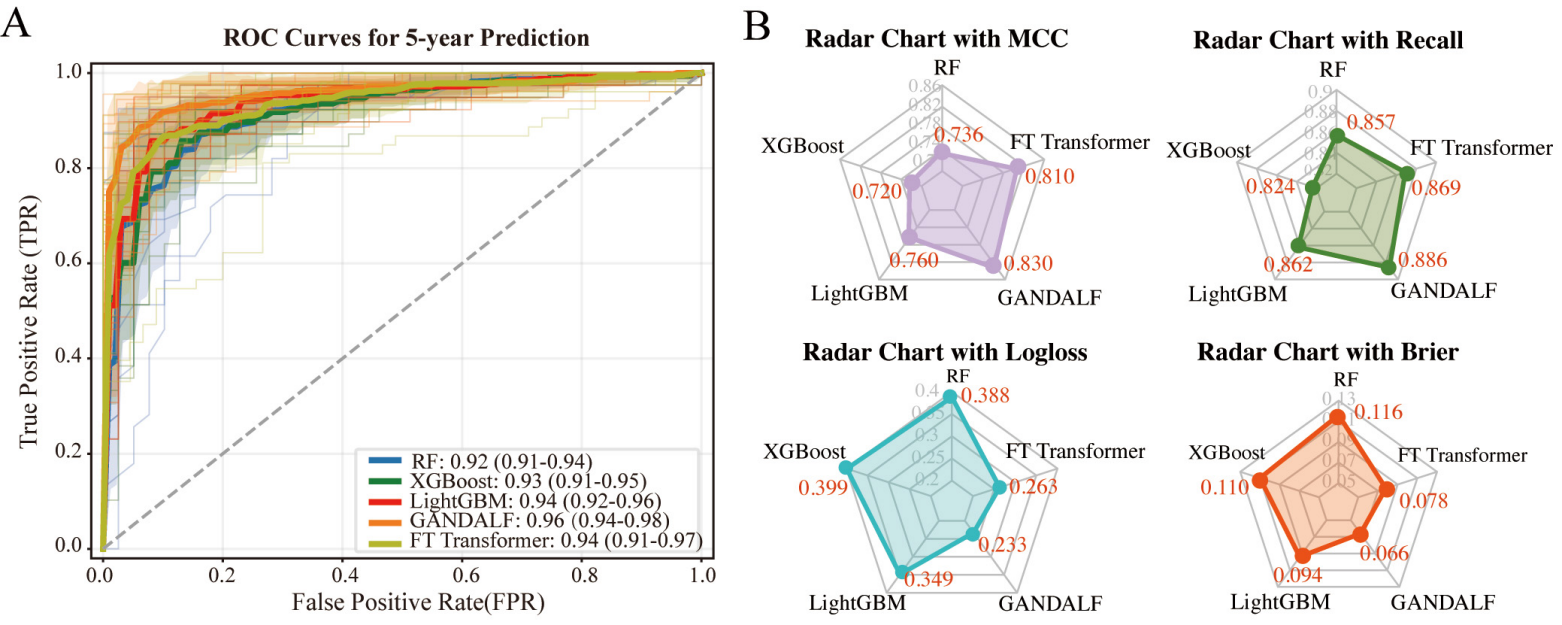
**Performance comparison of five machine learning and deep learning models for predicting depression in female patients with MAFLD**. (A) Comparison of ROC values for depression risk prediction models constructed using three machine learning models (Random Forest, XGBoost, LightGBM) and two deep learning models (FT Transformer, Gated Adaptive Network for Deep Automated Learning of Features [GANDALF]) in the MAFLD population. (B) Radar charts showing the Matthews’ correlation coefficient (MCC), Recall, Brier score, and log-loss values for the five models.

We next incorporated the 15 most predictive variables, along with the two sex-specific factors, into five machine learning/deep learning (ML/DL) models to evaluate five-year depression incidence. The performance of these models is shown in Fig. 4 (**Supplementary Table 4**). All models demonstrated exceptional predictive accuracy, with AUC values ranging from 0.92 to 0.96, underscoring their robust discriminative ability in distinguishing female participants with and without depression over the follow-up period.

Notably, the deep learning framework GANDALF exhibited superior performance across multiple evaluation metrics compared to the most commonly used ML models in clinical settings. It achieved an accuracy of 0.914 ± 0.039, an AUC of 0.96, and an MCC of 0.830 ± 0.068, highlighting its efficacy in handling imbalanced datasets. In terms of sensitivity and specificity, GANDALF achieved scores of 0.886 ± 0.064 and 0.942 ± 0.023, respectively, indicating its ability to accurately identify both affected and unaffected individuals. Moreover, the high F1 score (0.910 ± 0.039) and recall (0.886 ± 0.061) further attest to its strong sensitivity to true positives, while maintaining balanced precision. Beyond classification accuracy, GANDALF showed particularly strong calibration properties, as evidenced by its low Brier score (0.066 ± 0.025) and Log-Loss (0.233 ± 0.078). The three conventional ML approaches also demonstrated comparable efficacy, with MCC values ranging from 0.720 to 0.760, ROC-AUC from 0.92 to 0.94, recall from 0.824 to 0.862, F1 scores from 0.852 to 0.876, accuracy from 0.858 to 0.878, Brier scores from 0.094 to 0.116, and Log-Loss from 0.349 to 0.399.

Based on this comprehensive evaluation, GANDALF was selected as the optimal model for predicting depression. The optimal hyperparameters, identified through grid search, are detailed in **Supplementary Table 5**.

### 3.7 Interpretable Machine Learning

**Fig. 5.**
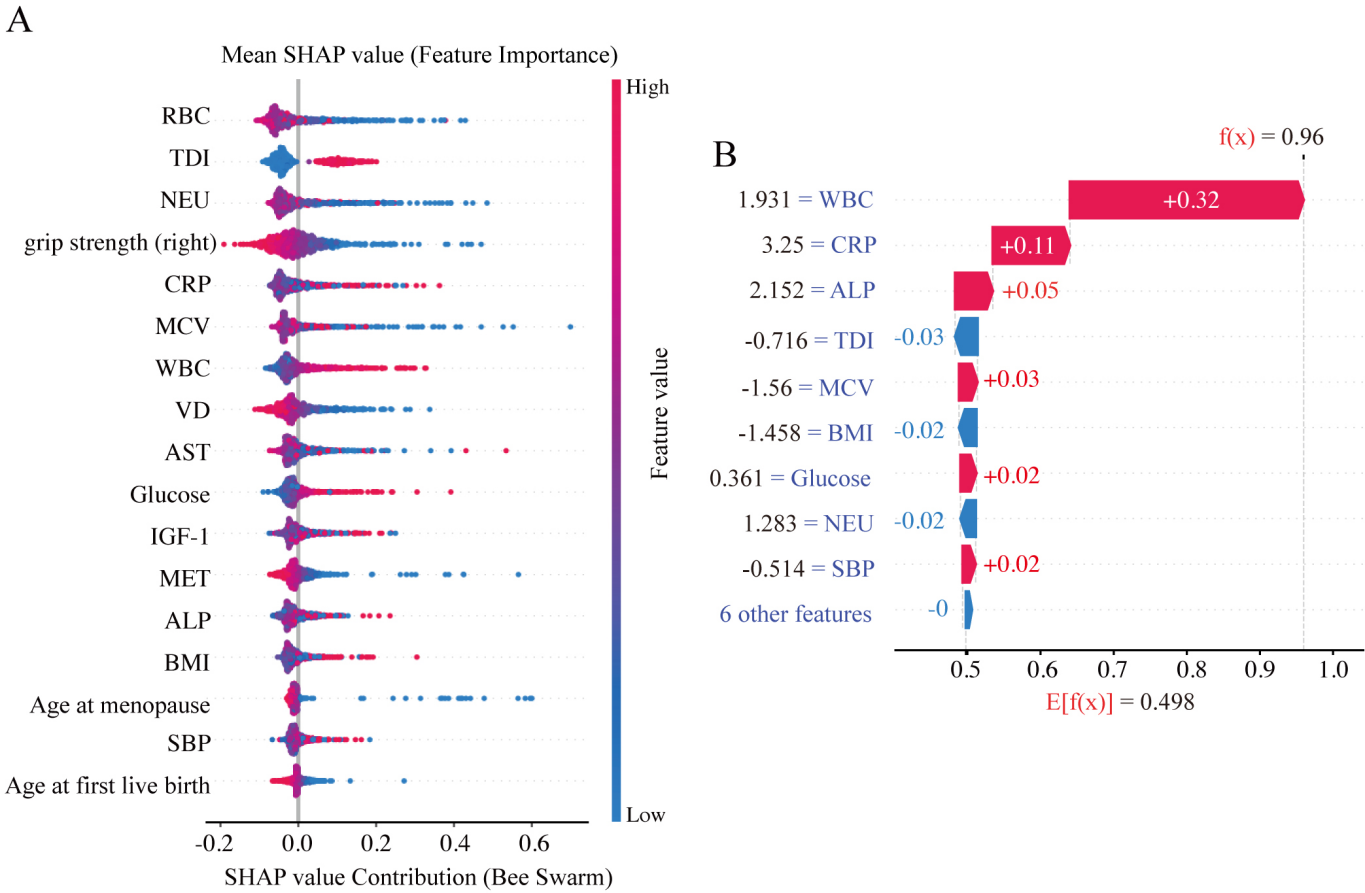
**Global and local model explanation using the SHapley Additive exPlanation (SHAP) method**. (A) SHAP summary dot plot. The top X-axis shows the mean absolute SHAP value for each feature, indicating their overall importance, while the bottom X-axis displays the distribution of SHAP values across all samples. Each dot represents an individual’s SHAP value for a given feature, with red indicating higher feature values and blue indicating lower ones. Vertical stacking of dots reflects data density. (B) SHAP waterfall plot for the ninth patient, illustrating the contribution of each feature to the final prediction made by the GANDALF model. Red bars denote features that positively contribute to the prediction, while blue bars indicate features with negative contributions.

The SHAP analysis offered a robust interpretability framework for our predictive model, enabling both global and local insights into the contribution of features. At the global level, the SHAP summary plot (Fig. 5A) visualizes the distribution of SHAP values for each feature across the dataset. Each dot represents a single data point, with the lower X-axis denoting the distribution of SHAP values and the upper X-axis showing the mean absolute SHAP value for each feature, which serves as an indicator of feature importance. Among the predictors of depression risk in female patients with MAFLD, the five most influential, ranked by their mean SHAP values, were red blood cell count (RBC), Townsend deprivation index (TDI), neutrophil count (NEU), right grip strength, and C-reactive protein (CRP). Specifically, lower RBC counts, elevated TDI values, reduced NEU levels, increased white blood cell counts (WBC), diminished physical activity, and higher glucose levels were consistently associated with an elevated risk of comorbid depression in this population. These findings not only enhance the transparency of the model, but also pinpoint modifiable clinical risk factors that could guide targeted interventions.

To further facilitate individualized clinical decision-making, the utility of SHAP analysis was extended by providing localized interpretations of how specific feature values influenced the predicted depression risk for each patient. A personalized risk score for depression in MAFLD patients was derived using the following formula:

$${\text{ SHAP Value }}_{i}=b_{i}\,\left(x_{i}\right)\cdot \pi \,\left(x_{i}\right)$$

$$f\,\left(x_{i}\right)=f_{\text{base }}\,\left(x\right)+\sum_{i=1}^{n}\left({\text{ SHAP Value }}_{i}\cdot {\text{ Feature Value }}_{i}\right)$$

where

• $f_{b\,a\,s\,e}\,\left(x\right)$ represents the average value of the target variable across all samples;

• $b_{i}$represents the regression coefficient for feature I;

• $\pi \,\left(x_{i}\right)$ signifies the baseline feature value for $x_{i}$.

The representative case shown in Fig. 5B highlights this approach. For this individual, WBC and CRP emerged as the most significant positive contributors to depression risk, with SHAP values of +0.32 and +0.11, respectively. Conversely, a lower TDI and BMI exerted a protective effect, each contributing a SHAP value of –0.03 and –0.02. This analysis culminated in a total personalized risk score of f⁢(x)=0.96, underscoring the model’s capacity to produce patient-specific risk estimates.

### 3.8 Nonlinear Effects of Key Predictors on Depression Risk

**Fig. 6.**
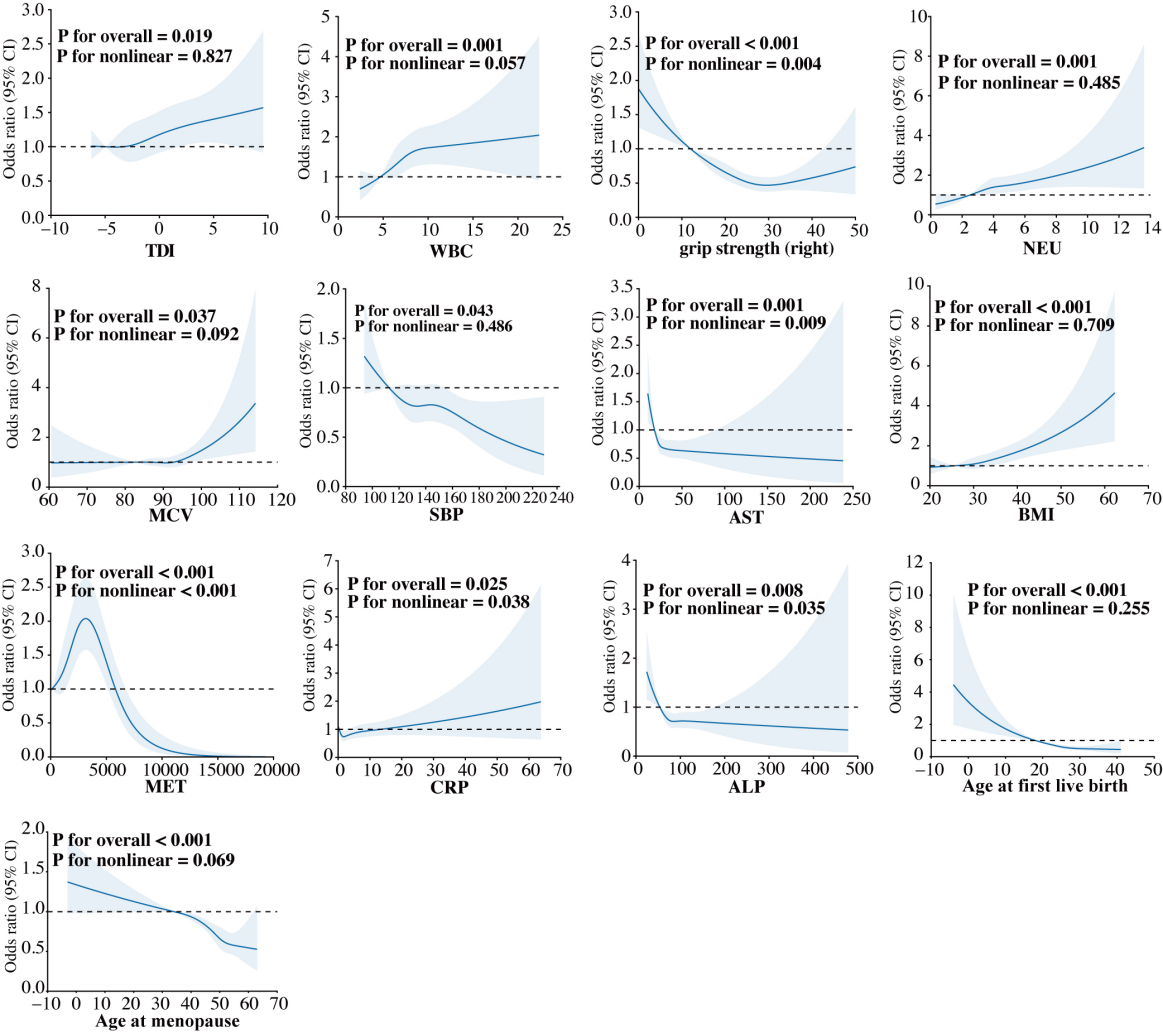
**Restricted cubic splines (RCS) curves explore the nonlinear relationships between risk factors and depression risk, offering deeper insights into complex associations**.

RCS analysis quantified the effects of key risk factors on the likelihood of comorbid depression and provided evidence for nonlinear associations. As shown in Fig. 6, notable nonlinear relationships were identified between several predictors (e.g., right hand-grip strength, aspartate aminotransferase [AST], summed MET minutes, CRP, alkaline phosphatase [ALP]) and depression risk, with statistical significance (*p* < 0.05) established for both overall and nonlinear effects.

Particularly striking was the RCS curve for summed MET minutes, which demonstrated a complex nonlinear pattern (*p* < 0.001 for both overall and nonlinear effects). Contrary to a simple protective effect, the risk appeared to peak at moderate activity levels (around 3000 MET minutes/week) before declining significantly at higher intensities (>5000 MET minutes/week). A distinct trend was observed for right-hand grip strength. While lower levels of grip strength correlated with elevated depression risk, the risk diminished progressively beyond a threshold of 10 kg. Additionally, elevated BMI (>25 kg/m^2^), WBC (>5 × 10^9^/L), and NEU (>3 × 10^9^/L) were positively associated with elevated depression risk, positioning these variables as potential markers of vulnerability in MAFLD patients. Similarly, the TDI showed a linear positive association (*p* for nonlinear = 0.827), where the risk of depression escalated with increasing deprivation scores, reaching its peak at a score of 10. Furthermore, earlier age at first live birth (<20 years) and menopause (<35 years) among female participants were consistently linked to an increased risk of depression. These findings offer valuable clinical insights and may inform more personalized and targeted intervention strategies.

### 3.9 Comparative Validation of Feature Specificity in Female Populations

To ascertain the specificity and robustness of the selected features for predicting depression in females with MAFLD, we conducted a comparative evaluation against established predictor sets from prior studies. Specifically, we incorporated 29 depression predictors identified by Radford-Smith *et al*. [28] (comprising psychological factors, blood cell counts, and physical measures) and 20 features previously reported by Ma *et al*. [29] (focusing primarily on plasma metabolites and lifestyle factors) into the GANDALF model, alongside our own curated feature set. For each model, 20% of the data was withheld for independent testing, while the remaining 80% underwent ten iterations of 10–fold cross-validation.

The results, detailed in **Supplementary Table 6**, indicate that our selected features outperformed those of Radford-Smith* et al*. [28] (ROC-AUC: 0.886, Accuracy: 0.825, MCC: 0.654, F1: 0.816, Recall: 0.789, Brier: 0.125, Log-Loss: 0.401) and those of Ma* et al*. [29] (ROC-AUC: 0.940, Accuracy: 0.905, MCC: 0.813, F1: 0.894, Recall: 0.844, Brier: 0.079, Log-Loss: 0.275). The superior performance of our feature set, tailored to female MAFLD patients, suggests that it can significantly improve the accuracy of depression prediction within this demographic.

## 4. Discussion

This study developed a lightweight, yet highly accurate model for the prediction of depression risk that leverages reduced feature dimensionality and was tailored specifically to female patients with MAFLD. We leveraged a comprehensive dataset of lifestyle indices, biochemical markers, routine blood test parameters, and sex-specific factors from the large-scale UK Biobank cohort. We then applied logistic regression—adjusted for sociodemographic, lifestyle, comorbidities, and medication use—and rigorous, data-driven feature selection to identify 17 key predictors. The GANDALF model achieved an impressive ROC-AUC of 0.96 ± 0.03 for predicting depression over five years, surpassing the predictive performance of feature sets from two prior studies in comparative analyses. A key strength of this model lies in its capacity to generate individualized depression risk scores, offering significant potential for personalized treatment planning and targeted early interventions. By centering on sex-specific risk factors, this approach enhances early detection and facilitates tailored preventive strategies, significantly improving mental health outcomes for women with MAFLD who have a high depression risk.

In the experimental evaluation, we systematically assessed the predictive performance of two DL models—GANDALF and FT-Transformer—alongside three traditional ML algorithms. The GANDALF and FT-Transformer models, rooted in the Gated Feature Learning Unit (GFLU) and Transformer architecture, respectively, notably outperformed traditional ML methods. This finding challenges the conventional reliance on ML techniques for tabular prediction tasks and underscores the promise of modern DL architectures when properly adapted to the structural nuances of tabular data.

The superior performance of GANDALF in our study can be attributed to its unique architectural advantages that address the specific challenges of clinical tabular data. Unlike traditional Multilayer Perceptrons (MLPs) that process all input features globally, GANDALF employs Gated Feature Learning Units (GFLUs) equipped with a t-softmax mechanism [18]. This mechanism functions in a similar manner to feature selection in tree-based models, effectively filtering out noisy or irrelevant clinical indicators. This is a critical capability given the high dimensionality and noise often present in medical datasets. Furthermore, GANDALF transcends the limitations of Gradient Boosted Decision Trees (GBDTs). While GBDTs rely on hard decision boundaries, GANDALF utilizes a differentiable gating mechanism (inspired by GRUs) to capture complex, non-linear interactions between metabolic markers and depression risk [18]. This allows smoother decision boundaries and robust hierarchical representation learning, enabling the model to identify subtle physiological patterns that might be missed by simpler models. Similarly, the FT-Transformer harnesses multi-head, self-attention mechanisms to dynamically construct correlation weight matrices across input features, thereby facilitating context-aware and adaptive feature interactions based on global semantic relationships [19]. This attention-driven strategy enables the creation of intelligent, task-relevant feature combinations that account for underlying contextual dependencies, offering a sophisticated approach to modeling the intricate health profiles of female MAFLD patients.

The feature selection phase was strengthened by multiple stratified bootstrap resampling, ensuring balanced distributions of key covariates—such as age and BMI—across experimental and control groups. By iteratively combining these techniques, our approach effectively eliminated confounding factors while isolating disease-specific biomarkers. This approach significantly enhanced the robustness and biological interpretability compared to conventional single-method frameworks.

Beyond model evaluation, our analysis yielded novel insights into the impact of female reproductive factors on depression risk. Notably, earlier ages at first live birth and menopause emerged as significant contributors to elevated depression risk, aligning with broader epidemiological evidence. For instance, a recent cross-sectional study of 1260 individuals identified early motherhood as a distinct vulnerability factor for depression [30]. Similarly, Bottino *et al*. [31] reported that among 811 postpartum mothers, every one-year delay in pregnancy reduced the likelihood of postpartum depression by 4%. This association was independent of socioeconomic status. Reports in the literature suggest that hormonal and neurobiological mechanisms may mediate this risk. The rapid postpartum decline in allopregnanolone might induce depression by altering the activity of gamma-aminobutyric acid receptor [32], with younger mothers experiencing a more pronounced reduction and hence increased susceptibility. Furthermore, early childbirth may disrupt the hypothalamic-pituitary-adrenal (HPA) axis before full physiological maturation, thereby elevating cortisol levels. This risk factor is likely to be exacerbated by the metabolic stress inherent to MAFLD [33, 34].

Similar patterns emerged regarding menopause. A large retrospective study confirmed that earlier menopause is associated with increased depression risk [35], with multifactorial, hormonal dynamics likely to play a plausible role. Premature menopause (<35 years), often reflective of premature ovarian insufficiency, involves a sharp reduction in the estrogen level. Since estrogen exerts neuroprotective effects by modulating serotonin and dopamine pathways, its premature depletion may impair these systems [36]. In MAFLD, the loss of estrogen’s anti-inflammatory properties could potentially exacerbate hippocampal neuronal damage, further predisposing individuals to depression [37].

The complexity of depression risk in MAFLD extends to modifiable lifestyle and metabolic domains, where our RCS analysis revealed distinct non-linear dose-response relationships. The “U-shaped” association for physical activity (summed MET minutes) is particularly intriguing. Moderate physical activity (0–6000 MET minutes/week) exhibited a robust protective effect, plausibly mediated by endorphins and BDNF [38, 39]. However, excessive activity was paradoxically associated with elevated risk. This observation aligns with the biological concept of hormesis, where beneficial stressors become maladaptive at high doses [40]. For MAFLD patients with underlying metabolic fragility, excessive exertion may induce “overtraining syndrome”, leading to chronic cortisol elevation and systemic oxidative stress [41, 42]. This physiological strain can aggravate pre-existing low-grade inflammation, potentially overwhelming the neuroprotective benefits of exercise.

Complementing these findings, right-hand grip strength followed a distinct threshold pattern, highlighting the role of skeletal muscle as an endocrine organ. Muscle tissue secretes neuroprotective myokines (e.g., irisin and IL-6) [43, 44]. The elevated depression risk at lower grip strength likely reflects sarcopenia or sarcopenic obesity. Below a critical threshold of approximately 10 kg, the deficiency in myokines, compounded by the burden of frailty, may precipitate depression. Conversely, once there is sufficient muscle strength to support metabolic health, further increases yield diminishing returns, explaining the plateau observed in our RCS curves.

Our study further identified routine hematological parameters as critical systemic predictors. Notably, elevated RBC counts were associated with a reduced risk of depression. This protective effect may be attributed to enhanced oxygen-carrying capacity, potentially alleviating cerebral hypoxic stress—a condition implicated in depression pathophysiology [45, 46]. Conversely, elevated WBC counts were linked to an increased risk of depression, providing compelling evidence for a pro-inflammatory mechanism. WBCs, particularly neutrophils and lymphocytes, serve as key mediators of systemic inflammation, with elevated levels often signaling an activated immune response [47, 48]. In the context of MAFLD, chronic low-grade inflammation may stimulate WBC proliferation and the release of pro-inflammatory cytokines (e.g., IL-6, TNF-α) [49, 50]. These cytokines can cross the blood-brain barrier, promoting neuroinflammation and disrupting neurotransmitter balance, thereby fostering depressive symptoms.

The limitations of our study include three key aspects. First, the study cohort was predominantly composed of middle-aged, white British individuals with a mean age of approximately 58 years, thus limiting the generalizability of our findings to more diverse populations. Future investigations should validate the model across broader ethnicities and age groups. Second, the inclusion of antidepressant use warrants cautious interpretation. Although this strategy was necessary to preserve statistical power and control for metabolic confounding, it may also capture prodromal depressive states, potentially contributing to the model’s high discriminative performance. Future investigations with larger sample sizes should consider sensitivity analyses that strictly exclude baseline medication users to further substantiate these findings. Finally, regarding clinical deployability, while our model utilizes routinely available input features (e.g., blood biochemistry and demographics), the computational infrastructure required for deep learning inference may pose challenges in resource-limited primary care settings. To address this, future implementation strategies should prioritize seamless integration with Electronic Health Record systems to streamline data collection and reduce technical barriers.

## 5. Conclusions

In conclusion, this study established the GANDALF-based deep learning framework as a superior diagnostic instrument. Its remarkable AUC of 0.96 significantly outperforms traditional machine learning algorithms. By integrating 17 critical features—including lifestyle indices, biochemical markers, routine blood test parameters, and sex-specific factors—our model is capable of unraveling the complex, non-linear determinants of depression in MAFLD patients. Moreover, it provides a robust, precision-medicine tool for early risk stratification and targeted intervention in this vulnerable population.

## Availability of Data and Materials

The datasets analyzed during the current study are available from the UK Biobank (https://www.ukbiobank.ac.uk/) upon successful project application and data access agreement. The data used in this study were obtained under Application Number 99946.

## Supplementary Material

Supplementary material associated with this article can be found, in the online version, at https://doi.org/10.31083/AP46337.

## References

[1] Le MH, Yeo YH, Zou B, Barnet S, Henry L, Cheung R (2022). Forecasted 2040 global prevalence of nonalcoholic fatty liver disease using hierarchical bayesian approach. *Clinical and Molecular Hepatology*.

[2] Eng PC, Forlano R, Tan T, Manousou P, Dhillo WS, Izzi-Engbeaya C (2023). Non-alcoholic fatty liver disease in women - Current knowledge and emerging concepts. *JHEP Reports: Innovation in Hepatology*.

[3] Byrne CD, Targher G (2015). NAFLD: a multisystem disease. *Journal of Hepatology*.

[4] Wang A, Tian X, Deng Q, Zheng M, Xia X, Zhang Y (2025). Longitudinal association of metabolic dysfunction-associated fatty liver disease, serum metabolites, with cognitive function trajectories. *npj Metabolic Health and Disease*.

[5] Bambha K, Belt P, Abraham M, Wilson LA, Pabst M, Ferrell L (2012). Ethnicity and nonalcoholic fatty liver disease. *Hepatology*.

[6] Kim D, Yoo ER, Li AA, Tighe SP, Cholankeril G, Harrison SA (2019). Depression is associated with non-alcoholic fatty liver disease among adults in the United States. *Alimentary Pharmacology & Therapeutics*.

[7] Xiao J, Lim LKE, Ng CH, Tan DJH, Lim WH, Ho CSH (2021). Is Fatty Liver Associated With Depression? A Meta-Analysis and Systematic Review on the Prevalence, Risk Factors, and Outcomes of Depression and Non-alcoholic Fatty Liver Disease. *Frontiers in Medicine*.

[8] Di Benedetto MG, Landi P, Mencacci C, Cattaneo A (2024). Depression in Women: Potential Biological and Sociocultural Factors Driving the Sex Effect. *Neuropsychobiology*.

[9] McCormick CM, Mathews IZ (2007). HPA function in adolescence: role of sex hormones in its regulation and the enduring consequences of exposure to stressors. *Pharmacology, Biochemistry, and Behavior*.

[10] Dong J, Dennis KMJH, Venkatakrishnan R, Hodson L, Tomlinson JW (2025). The Impact of Estrogen Deficiency on Liver Metabolism: Implications for Hormone Replacement Therapy. *Endocrine Reviews*.

[11] Chen JM, Rao M, Wei YT, Zhou QG, Tao JL, Wang SB (2025). Machine learning-based nomogram for predicting depressive symptoms in women: A cross-sectional study in Guangdong Province, China. *World Journal of Psychiatry*.

[12] Sudlow C, Gallacher J, Allen N, Beral V, Burton P, Danesh J (2015). UK biobank: an open access resource for identifying the causes of a wide range of complex diseases of middle and old age. *PLoS Medicine*.

[13] Collins R (2007). UK Biobank: Protocol for a large-scale prospective epidemiological resource. https://www.ukbiobank.ac.uk/wp-content/uploads/2025/01/Main-study-protocol.pdf.

[14] Wryk G, Gawor A, Bulska E (2024). Comprehensive Evaluation of Advanced Imputation Methods for Proteomic Data Acquired via the Label-Free Approach. *International Journal of Molecular Sciences*.

[15] Feng C, Wang H, Lu N, Chen T, He H, Lu Y (2014). Log-transformation and its implications for data analysis. *Shanghai Archives of Psychiatry*.

[16] Ke G, Meng Q, Finley T, Wang T, Chen W, Ma W, Ye Q, Liu TY (2017). Lightgbm: A highly efficient gradient boosting decision tree. *In Advances in Neural Information Processing Systems 30*.

[17] You J, Zhang YR, Wang HF, Yang M, Feng JF, Yu JT (2022). Development of a novel dementia risk prediction model in the general population: A large, longitudinal, population-based machine-learning study. *EClinicalMedicine*.

[18] Joseph M, Raj H (2022). GANDALF: gated adaptive network for deep automated learning of features. *arXiv*.

[19] Jaffari ZH, Abbas A, Kim CM, Shin J, Kwak J, Son C (2024). Transformer-based deep learning models for adsorption capacity prediction of heavy metal ions toward biochar-based adsorbents. *Journal of Hazardous Materials*.

[20] Yen SJ, Lee YS (2009). Cluster-based under-sampling approaches for imbalanced data distributions. *Expert Systems with Applications*.

[21] Kartini D, Nugrahadi DT, Farmadi A (2021). Hyperparameter tuning using GridsearchCV on the comparison of the activation function of the ELM method to the classification of pneumonia in toddlers. *In 2021 4th international conference of computer and informatics engineering (IC2IE)*.

[22] Rufibach K (2010). Use of Brier score to assess binary predictions. *Journal of Clinical Epidemiology*.

[23] Nangir M, Asvadi R, Ahmadian-Attari M, Chen J (2018). Analysis and code design for the binary CEO problem under logarithmic loss. *IEEE Transactions on Communications*.

[24] Chicco D, Jurman G (2020). The advantages of the Matthews correlation coefficient (MCC) over F1 score and accuracy in binary classification evaluation. *BMC Bioinformatics*.

[25] Antwarg L, Miller RM, Shapira B, Rokach L (2021). Explaining anomalies detected by autoencoders using Shapley Additive Explanations. *Expert Systems with Applications*.

[26] Bedogni G, Bellentani S, Miglioli L, Masutti F, Passalacqua M, Castiglione A (2006). The Fatty Liver Index: a simple and accurate predictor of hepatic steatosis in the general population. *BMC Gastroenterology*.

[27] Liu Y, Wang J, Jin R, Xu Z, Zhao X, Li Y (2024). Associations of Metabolic Dysfunction-Associated Fatty Liver Disease With Peripheral Artery Disease: Prospective Analysis in the UK Biobank and ARIC Study. *Journal of the American Heart Association*.

[28] Radford-Smith DE, Anthony DC, Benz F, Grist JT, Lyman M, Miller JJ (2023). A multivariate blood metabolite algorithm stably predicts risk and resilience to major depressive disorder in the general population. *EBioMedicine*.

[29] Ma S, Xie X, Deng Z, Wang W, Xiang D, Yao L (2024). A Machine Learning Analysis of Big Metabolomics Data for Classifying Depression: Model Development and Validation. *Biological Psychiatry*.

[30] Kolahdooz A, Movahed F, Yousefi M, Salehi A, Goodarzi S, Shafiee A (2025). The Association Between Age at First Live Birth and Depression: Results From NHANES 2005-2018. *Depression and Anxiety*.

[31] Bottino MN, Nadanovsky P, Moraes CL, Reichenheim ME, Lobato G (2012). Reappraising the relationship between maternal age and postpartum depression according to the evolutionary theory: Empirical evidence from a survey in primary health services. *Journal of Affective Disorders*.

[32] Deligiannidis KM, Sikoglu EM, Shaffer SA, Frederick B, Svenson AE, Kopoyan A (2013). GABAergic neuroactive steroids and resting-state functional connectivity in postpartum depression: a preliminary study. *Journal of Psychiatric Research*.

[33] McCormick CM, Mathews IZ (2010). Adolescent development, hypothalamic-pituitary-adrenal function, and programming of adult learning and memory. *Progress in Neuro-psychopharmacology & Biological Psychiatry*.

[34] Rinne GR, Hartstein J, Guardino CM, Dunkel Schetter C (2023). Stress before conception and during pregnancy and maternal cortisol during pregnancy: A scoping review. *Psychoneuroendocrinology*.

[35] Kim H, Jung JH, Han K, Lee DY, Fava M, Mischoulon D (2023). Ages at menarche and menopause, hormone therapy, and the risk of depression. *General Hospital Psychiatry*.

[36] Joffe H, Cohen LS (1998). Estrogen, serotonin, and mood disturbance: where is the therapeutic bridge?. *Biological Psychiatry*.

[37] Tauil RB, Golono PT, de Lima EP, de Alvares Goulart R, Guiguer EL, Bechara MD (2024). Metabolic-Associated Fatty Liver Disease: The Influence of Oxidative Stress, Inflammation, Mitochondrial Dysfunctions, and the Role of Polyphenols. *Pharmaceuticals*.

[38] Wang CS, Kavalali ET, Monteggia LM (2022). BDNF signaling in context: From synaptic regulation to psychiatric disorders. *Cell*.

[39] Zarza-Rebollo JA, López-Isac E, Rivera M, Gómez-Hernández L, Pérez-Gutiérrez AM, Molina E (2024). The relationship between BDNF and physical activity on depression. *Progress in Neuro-psychopharmacology & Biological Psychiatry*.

[40] Militello R, Luti S, Gamberi T, Pellegrino A, Modesti A, Modesti PA (2024). Physical Activity and Oxidative Stress in Aging. *Antioxidants*.

[41] Kraemer WJ, Ratamess NA (2005). Hormonal responses and adaptations to resistance exercise and training. *Sports Medicine*.

[42] Radak Z, Chung HY, Koltai E, Taylor AW, Goto S (2008). Exercise, oxidative stress and hormesis. *Ageing Research Reviews*.

[43] Severinsen MCK, Pedersen BK (2020). Muscle-Organ Crosstalk: The Emerging Roles of Myokines. *Endocrine Reviews*.

[44] Muñoz-Cánoves P, Scheele C, Pedersen BK, Serrano AL (2013). Interleukin-6 myokine signaling in skeletal muscle: a double-edged sword?. *The FEBS Journal*.

[45] Jensen FB (2009). The dual roles of red blood cells in tissue oxygen delivery: oxygen carriers and regulators of local blood flow. *The Journal of Experimental Biology*.

[46] Hess AS (2024). Oxygen Extraction Ratios to Guide Red Blood Cell Transfusion. *Transfusion Medicine Reviews*.

[47] Abdulkhaleq LA, Assi MA, Abdullah R, Zamri-Saad M, Taufiq-Yap YH, Hezmee MNM (2018). The crucial roles of inflammatory mediators in inflammation: A review. *Veterinary World*.

[48] Xiong L, Fan C, Song J, Wan Y, Lin X, Su Z (2022). Associations of long-term cadmium exposure with peripheral white blood cell subtype counts and indices in residents of cadmium-polluted areas. *Chemosphere*.

[49] Rafaqat S, Gluscevic S, Mercantepe F, Rafaqat S, Klisic A (2024). Interleukins: Pathogenesis in Non-Alcoholic Fatty Liver Disease. *Metabolites*.

[50] Joo SK, Kim W (2023). Interaction between sarcopenia and nonalcoholic fatty liver disease. *Clinical and Molecular Hepatology*.

