# Supplementary material for: Prediction of Depression in Women With Metabolic Dysfunction-Associated Fatty Liver Disease Using Routine Blood Tests: A Five-Year Longitudinal Analysis From the UK Biobank
Source: Alpha Psychiatry. 2026 Jun 17;27(3):46337. doi: 10.31083/AP46337 (PMC13339881; doi:10.31083/AP46337)
Supplement: Supplementary file 1 [file 2757-8038-27-3-46337-s1.docx]

**Supplementary Materials**

**Supplementary Table 1.** List of blood biochemistry and blood cell count in the UK Biobank.

**Supplementary Table 2**. Mean±SD of blood biochemistry and routine blood test parameters in the UK Biobank.

**Supplementary Table 3.** Multinomial logistic regression models assessing the association between female-specific factors and depression prevalence.

**Supplementary Table 4**. Performance parameters of the 5 DL/ML prediction models in the test set.

**Supplementary Table 5**. Hyperparameters for 5 ML/DL models.

**Supplementary Table 6**. Comparative Analysis of Features from Prior Studies Evaluated in the GANDALF Model.

**Supplementary Fig. 1.** Sample Size and Power Analysis Result.

**Supplementary Fig. 2**. Spearman rank correlation heatmap of the final 15 selected predictors.

**Supplementary Text 1.** UK Biobank Data Collection Questionnaires

**Supplementary Table 1. List of blood biochemistry and blood cell count in the UK Biobank.**

| **Field id** | **Biomarker** | **Description** | **Field id** | **Biomarker** | **Description** | **Group** |  |
| --- | --- | --- | --- | --- | --- | --- | --- |
| p30610 | | Alkaline phosphatase | ALP | p30620 | Alanine aminotransferase | ALAT | Blood biochemistry |
| p30650 | | Aspartate aminotransferase | AST | p30670 | Urea | Urea |  |
| p30690 | | Cholesterol | CHOL | p23470 | Glucose | Glucose |  |
| p23478 | | Creatinine | Creatinine | p23479 | Albumin | ALB |  |
| p23439 | | Apolipoprotein B | ApoB | p23440 | Apolipoprotein A1 | ApoA1 |  |
| p23406 | | HDL Cholesterol | HDLC | p30780 | LDL direct | LDLd |  |
| p30790 | | Lipoprotein A | Lp(a) | p30850 | Testosterone | T |  |
| p30660 | | Direct bilirubin | DBil | p30680 | Calcium | Ca |  |
| p30710 | | C-reactive protein | CRP | p30720 | Cystatin C | CysC |  |
| p30730 | | Gamma glutamyltransferase | GGT | p30750 | Glycated haemoglobin (HbA1c) | HbA1c |  |
| p30770 | | IGF-1 | IGF-1 | p30800 | Oestradiol | E2 |  |
| p30810 | | Phosphate | Phosphate | p30860 | Total protein | TP |  |
| p30820 | | Rheumatoid factor | RF | p30870 | Triglycerides | TG |  |
| p30830 | | SHBG | SHBG | p30880 | Urate | Urate | Blood biochemistry |
| p30840 | | Total bilirubin | TBIL | p30890 | Vitamin D | VD |  |
| p30160 | | Basophill count | BAS | p30220 | Basophill percentage | BAS | Blood cell count |
| p30150 | | Eosinophill count | EOS | p30210 | Eosinophill percentage | EOS |  |
| p30030 | | Haematocrit percentage | HCT | p30020 | Haemoglobin concentration | Hb |  |
| p30300 | | High light scatter reticulocyte count | HLSR | p30290 | High light scatter reticulocyte percentage | HLSR |  |
| p30280 | | Immature reticulocyte fraction | IRF | p30120 | Lymphocyte count | LYM |  |
| p30180 | | Lymphocyte percentage | LYM | p30050 | Mean corpuscular haemoglobin | MCH |  |
| p30060 | | Mean corpuscular haemoglobin concentration | MCHC | p30040 | Mean corpuscular volume | MCV |  |
| p30100 | | Mean platelet (thrombocyte) volume | MPV | p30260 | Mean reticulocyte volume | MRV |  |
| p30270 | | Mean sphered cell volume | MSCV | p30130 | Monocyte count | Monocyte |  |
| p30190 | | Monocyte percentage | Monocyte | p30140 | Neutrophill count | NEU |  |
| p30200 | | Neutrophill percentage | NEU | p30170 | Nucleated red blood cell count | NRBC |  |
| p30230 | | Nucleated red blood cell percentage | NRBC | p30080 | Platelet count | PLT |  |
| p30090 | | Platelet crit | PCT | p30110 | Platelet distribution width | PDW | Blood cell count |
| p30010 | | Red blood cell (erythrocyte) count | RBC | p30070 | Red blood cell (erythrocyte) distribution width | RDW |  |
| p30250 | | Reticulocyte count | RET | p30240 | Reticulocyte percentage | RET |  |
| p30000 | | White blood cell (leukocyte) count | WBC |  |  |  |  |
|  | |  |  |  |  |  |  |

**Supplementary Table 2. Mean±SD of** **blood biochemistry and routine blood test parameters in the UK Biobank.**

| **biomarkers** | **Mean±SD** | **biomarkers** | **Mean±SD** |
| --- | --- | --- | --- |
| ALAT | 28.1**±**17.8 | Platelet count. | 271**±**60.9 |
| Albumin | 45.4**±**2.65 | Erythrocyte count | 4.41**±**0.34 |
| ALP | 87.6**±**28.5 | Leukocyte count | 7.25**±**1.94 |
| ApoA | 1.50**±**0.27 | Basophill count | 0.04**±**0.06 |
| ApoB | 1.07**±**0.25 | Eosinophill count | 0.18**±**0.14 |
| AST | 28.5**±**13.2 | Eosinophill percentage | 2.48**±**1.81 |
| CRP | 2.97**±**4.45 | Haematocrit percentage | 39.6**±**2.86 |
| Calcium | 2.39**±**0.09 | Haemoglobin concentration | 13.6**±**0.99 |
| Cholesterol | 5.82**±**1.20 | High light scatter reticulocyte count | 0.02**±**0.01 |
| Creatinine | 74.2**±**20.9 | High light scatter reticulocyte percentage | 0.49**±**0.75 |
| CysC | 0.93**±**0.19 | Immature reticulocyte fraction | 0.31**±**0.06 |
| DBIL | 1.83**±**0.90 | Lymphocyte percentage | 29.9**±**7.31 |
| GGT | 55.7**±**67.0 | Mean corpuscular haemoglobin | 31.0**±**2.02 |
| Glucose | 5.26**±**1.51 | Mean corpuscular haemoglobin concentration | 34.4**±**1.20 |
| HbA1c | 37.1**±**8.22 | Mean corpuscular volume | 90.0**±**4.73 |
| HDLC | 1.36**±**0.36 | Thrombocyte volume | 9.39**±**1.09 |
| IGF-1 | 21.1**±**5.75 | Mean reticulocyte volume | 105**±**7.90 |
| LDLd | 3.66**±**0.90 | Mean sphered cell volume | 82.0**±**5.12 |
| Phosphate | 1.15**±**0.17 | Monocyte count | 0.46**±**0.20 |
| SHBG | 45.0**±**25.1 | Monocyte percentage | 6.47**±**2.41 |
| Testosterone | 7.38**±**5.82 | Neutrophill count | 4.43**±**1.42 |
| TBIL | 9.11**±**4.42 | Neutrophill percentage | 60.6**±**8.23 |
| TP | 72.8**±**4.13 | Nucleated red blood cell count | 0.00**±**0.03 |
| TG | 2.28**±**1.31 | Nucleated red blood cell percentage | 0.04**±**0.45 |
| Urate | 331**±**83.1 | Platelet crit | 0.25**±**0.05 |
| Urea | 5.49**±**1.45 | Platelet distribution width | 16.4**±**0.51 |
| VD | 46.5**±**20.5 | Red blood cell (erythrocyte) distribution width | 13.6**±**1.07 |
| Reticulocyte percentage | 1.54**±**1.22 | Reticulocyte count | 0.07±0.04 |

**Supplementary Table 3.** Multinomial logistic regression models assessing the association between female-specific factors and depression prevalence.

| Description | Field ID | Model | OR | 95% CI Lower | 95% CI Upper | P-value |
| --- | --- | --- | --- | --- | --- | --- |
| Number of live births | p2734 | unadjusted model | 1.036 | 0.973 | 1.102 | 0.264 |
|  |  | model 1 | 1.036 | 0.973 | 1.102 | 0.264 |
|  |  | model 2 | 1.036 | 0.973 | 1.102 | 0.264 |
|  |  | model 3 | 1.020 | 0.958 | 1.087 | 0.521 |
| Age of primiparous women at birth of child | p3872 | unadjusted model | 0.992 | 0.960 | 1.024 | 0.616 |
|  |  | model 1 | 1.008 | 0.974 | 1.042 | 0.665 |
|  |  | model 2 | 1.013 | 0.979 | 1.048 | 0.451 |
|  |  | model 3 | 1.010 | 0.979 | 1.050 | 0.438 |
| Age at first live birth | p2754 | unadjusted model | 0.952 | 0.934 | 0.972 | 1.519 |
|  |  | model 1 | 0.968 | 0.947 | 0.989 | 0.003 |
|  |  | model 2 | 0.972 | 0.951 | 0.994 | 0.011 |
|  |  | model 3 | 0.973 | 0.952 | 0.995 | 0.015 |
| Age at last live birth | p2764 | unadjusted model | 0.984 | 0.967 | 1.001 | 0.064 |
|  |  | model 1 | 0.997 | 0.979 | 1.015 | 0.736 |
|  |  | model 2 | 0.998 | 0.980 | 1.017 | 0.836 |
|  |  | model 3 | 1.000 | 0.982 | 1.018 | 0.966 |
| Number of spontaneous miscarriages | p3839 | unadjusted model | 0.993 | 0.884 | 1.101 | 0.899 |
|  |  | model 1 | 0.995 | 0.888 | 1.100 | 0.932 |
|  |  | model 2 | 0.993 | 0.886 | 1.097 | 0.892 |
|  |  | model 3 | 0.983 | 0.877 | 1.090 | 0.752 |
| Age at menopause (last menstrual period) | p3581 | unadjusted model | 0.985 | 0.979 | 0.992 | 1.40E-05 |
|  |  | model 1 | 0.987 | 0.980 | 0.993 | 1.46E-04 |
|  |  | model 2 | 0.987 | 0.981 | 0.994 | 2.70E-04 |
|  |  | model 3 | 0.987 | 0.981 | 0.994 | 2.70E-04 |
| Age when periods started (menarche) | p2714 | unadjusted model | 0.984 | 0.957 | 1.014 | 0.267 |
|  |  | model 1 | 0.993 | 0.966 | 1.023 | 0.627 |
|  |  | model 2 | 0.993 | 0.965 | 1.023 | 0.609 |
|  |  | model 3 | 0.992 | 0.965 | 1.023 | 0.610 |
| Length of menstrual cycle | p3710 | unadjusted model | 0.993 | 0.982 | 1.005 | 0.257 |
|  |  | model 1 | 0.997 | 0.985 | 1.008 | 0.569 |
|  |  | model 2 | 0.996 | 0.985 | 1.010 | 0.564 |
|  |  | model 3 | 0.997 | 0.985 | 1.010 | 0.609 |
| Age at hysterectomy | p2824 | unadjusted model | 0.981 | 0.969 | 0.994 | 3.12E-03 |
|  |  | model 1 | 0.987 | 0.975 | 1.001 | 0.066 |
|  |  | model 2 | 0.989 | 0.976 | 1.003 | 0.119 |
|  |  | model 3 | 0.990 | 0.977 | 1.005 | 0.164 |
| Age started hormone-replacement therapy (HRT) | p3536 | unadjusted model | 0.990 | 0.984 | 0.997 | 3.41E-03 |
|  |  | model 1 | 0.993 | 0.987 | 1.000 | 0.050 |
|  |  | model 2 | 0.993 | 0.987 | 1.001 | 0.062 |
|  |  | model 3 | 0.994 | 0.987 | 1.001 | 0.092 |
| Age started oral contraceptive pill | p2794 | unadjusted model | 0.969 | 0.956 | 0.982 | 5.949E-06 |
|  |  | model 1 | 0.973 | 0.960 | 0.986 | 7.490E-05 |
|  |  | model 2 | 0.975 | 0.962 | 0.989 | 2.714E-04 |
|  |  | model 3 | 0.975 | 0.962 | 0.989 | 4.062E-04 |
| Age at bilateral oophorectomy (both ovaries removed) | p3882 | unadjusted model | 0.983 | 0.967 | 1.001 | 0.050 |
|  |  | model 1 | 0.988 | 0.972 | 1.008 | 0.203 |
|  |  | model 2 | 0.989 | 0.972 | 1.009 | 0.239 |
|  |  | model 3 | 0.988 | 0.971 | 1.008 | 0.193 |

Three progressively adjusted multinomial logistic regression models were employed:

Model 1: Adjusted for core sociodemographic covariates (age, ethnicity, educational attainment, BMI, household income);

Model 2: Model 1 + lifestyle factors (smoking status, alcohol consumption frequency, sleep quality, physical activity level);

Model 3: Model 2 + major comorbidities (hypertension, diabetes mellitus, cancer history).

Abbreviations: OR, odds ratio; CI, confidence interval.

**Supplementary Table 4. Performance parameters of the 5 DL/ML prediction models in the test set**

| **Model** | **Accuracy** | **ROC-AUC** | **F1** | **Recall** | **MCC** | **Sensitivity** | **Specificity** | **Brier** | **Logloss** |
| --- | --- | --- | --- | --- | --- | --- | --- | --- | --- |
| GANDALF | 0.914 | 0.957 | 0.911 | 0.886 | 0.830 | 0.886 | 0.942 | 0.066 | 0.233 |
| FT Transformer | 0.903 | 0.943 | 0.899 | 0.869 | 0.810 | 0.869 | 0.935 | 0.078 | 0.263 |
| Random Forest | 0.867 | 0.921 | 0.866 | 0.857 | 0.736 | 0.857 | 0.877 | 0.116 | 0.388 |
| XGBoost | 0.858 | 0.928 | 0.852 | 0.824 | 0.720 | 0.824 | 0.892 | 0.110 | 0.399 |
| lightGBM | 0.878 | 0.942 | 0.876 | 0.862 | 0.760 | 0.862 | 0.895 | 0.094 | 0.349 |

**Supplementary Table 5. Hyperparameters for 5 ML/DL models.**

| **Model** | **Hyperparameter** | **Search Range** | **Selected Value** |
| --- | --- | --- | --- |
| Random Forest | max_depth | [10, 20, 30, None] | 20 |
|  | max_features | ['sqrt', 'log2', None] | log2 |
|  | min_samples_leaf | [1,2,4] | 1 |
|  | min_samples_split | [2,5,10,20] | 10 |
|  | n_estimators | [10,50,100,200] | 50 |
| XGBoost | max_depth | [10,50,100,200] | 5 |
|  | min_child_weight | [1, 3, 5, 7] | 3 |
|  | n_estimators | [10,50,100,200] | 100 |
| LightGBM | num_leaves | [31, 63, 127] | 31 |
|  | min_data_in_leaf | [20, 50, 100] | 20 |
|  | max_depth | [5, 7, 10] | 5 |
| GANDALF | batch_size | [512, 1024] | 1024 |
|  | gflu_stages | [3, 6, 9, 12] | 6 |
|  | gflu_feature_init_sparsity | [0.1, 0.3, 0.5, 0.7] | 0.3 |
|  | gflu_dropout | [0.0, 0.05, 0.1] | 0.05 |
|  | learning_rate | [1e-4, 1e-3, 5e-3] | 1e-3 |
| FT Transformer | input_embed_dim | [8, 16, 32, 64] | 32 |
|  | num_attn_blocks | [1, 2, 3] | 2 |
|  | num_heads | [2, 4] | 4 |

**Supplementary Table 6. Comparative Analysis of Features from Prior Studies Evaluated in the GANDALF Model**

| **Study** | **Feature** | **Accuracy** | **ROC-AUC** | **F1** | **Recall** | **MCC** | **Sensitivity** | **Specificity** | **Brier** | **Log-loss** |
| --- | --- | --- | --- | --- | --- | --- | --- | --- | --- | --- |
| My study | MCV + IGF-1 + CRP + NEU + Glucose + AST + TDI + WBC + RBC + VD + BMI + grip strength (right) + MET + ALP + SBP + age at first live birth + age at menopause | 0.914 | 0.957 | 0.911 | 0.886 | 0.830 | 0.886 | 0.942 | 0.066 | 0.233 |
| Daniel E. Radford-Smith’s study | Neuroticism + Basophil count + TDI + Glucose + Lactate + BMI + WBFM + Body fat + Pyruvate + Haematocrit + Haemoglobin conc. + RBC + Creatinine + TBIL + VD + Glycoprotein acetylation + Age + Testosterone + illness count + disability + chronic pain + gender | 0.825 | 0.886 | 0.816 | 0.789 | 0.654 | 0.789 | 0.864 | 0.125 | 0.401 |
| Simeng Ma’s study | Gender + age + smoking + chronic_num + deprivation + IPAQ + Pyruvate + Alcohol + Acetate + Glucose + BMI + Gly + Lactate + Citrate + Unsaturation + Creatinine + Leu + MUFA + LDL_size + PUFA_by_MUFA | 0.905 | 0.940 | 0.894 | 0.844 | 0.813 | 0.944 | 0.962 | 0.079 | 0.275 |

Abbreviations: RBC, Red blood cell count; TDI, Townsend deprivation index; NEU, Neutrophill count; CRP, C-reactive protein; MET, Summed MET (min); MCV, Mean corpuscular volume; WBC, White blood cell (leukocyte) count; VD, Vitamin D; AST, Aspartate aminotransferase; ALP, Alkaline phosphatase; SBP, Systolic blood pressure; TBIL, Total bilirubin; chronic_num, The number of chronic diseases; BMI, body mass index; IPAQ, the International Physical; Activity Questionnaire; Gly, glycine; Leu, leucine; MUFA, monounsaturated fatty acids to total fatty acids percentage; PUFA_by_MUFA, polyunsaturated fatty acids to monounsaturated fatty acids ratio.

**Supplementary Fig. 1. Sample Size and Power Analysis Result.**

**
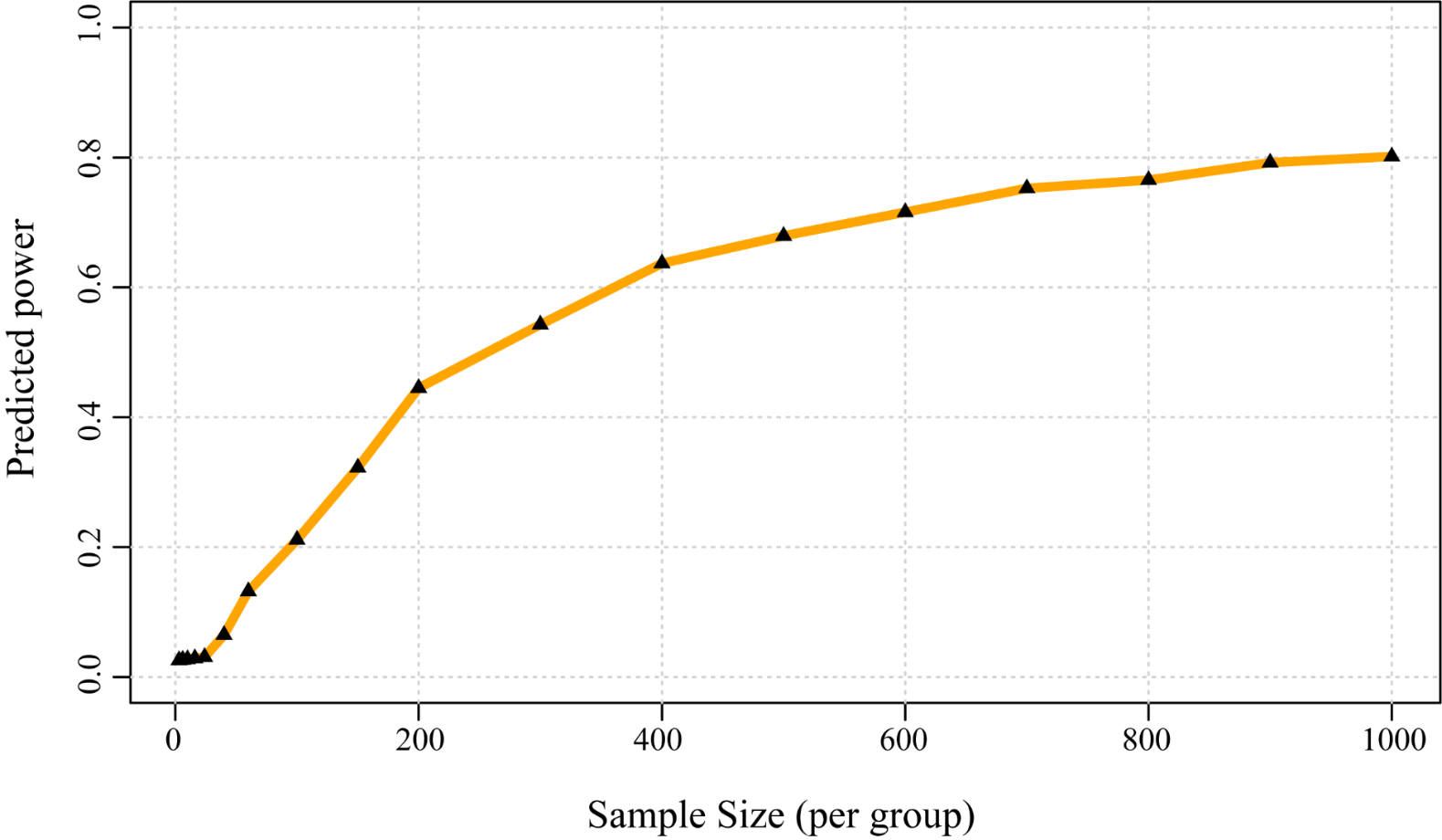
**

**Supplementary Fig. 2. Spearman rank correlation heatmap of the final 15 selected predictors.**

**
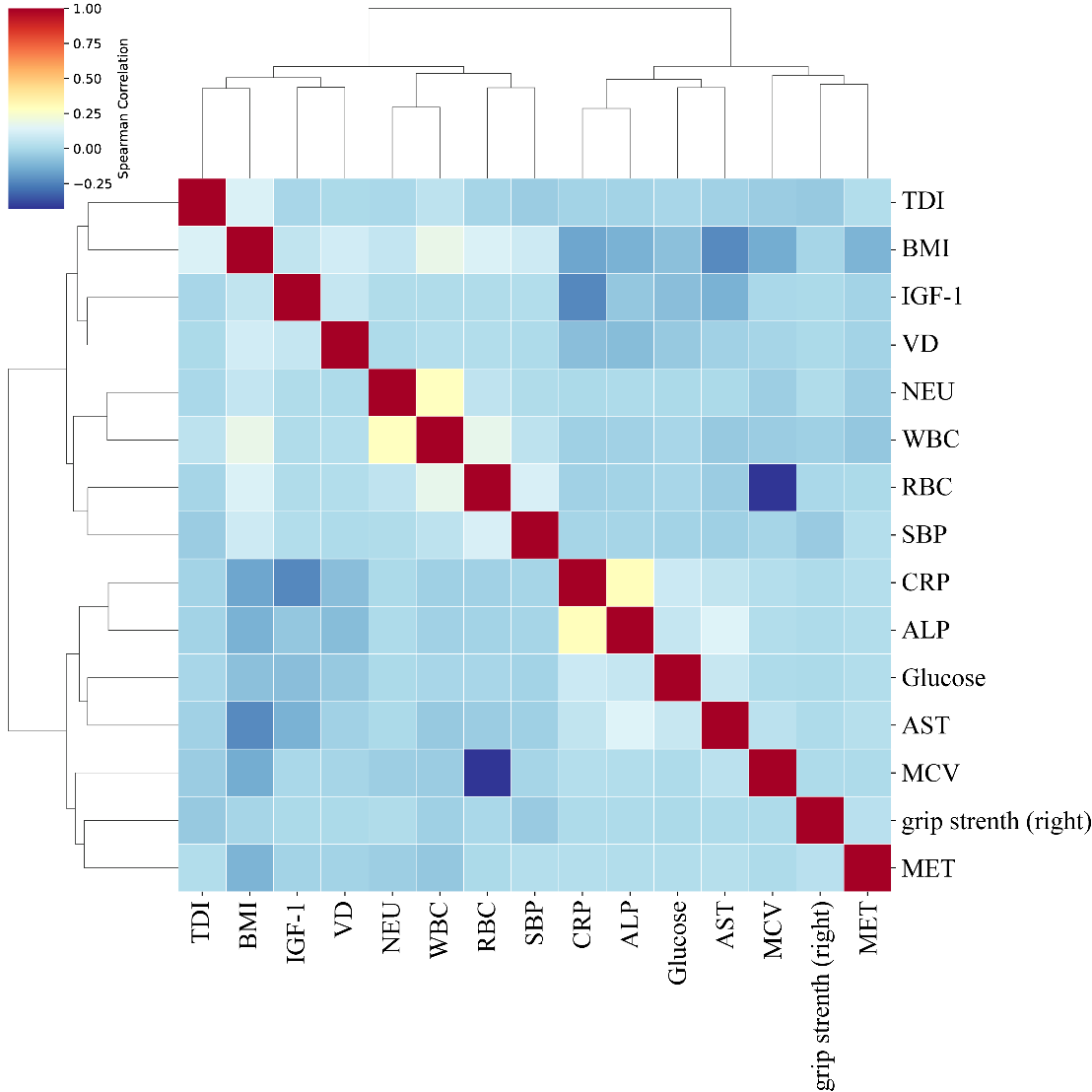
**

**Supplementary Text 1: UK Biobank Data Collection Questionnaires**

The data used in this study were collected via the UK Biobank assessment centre. Participants completed a touchscreen questionnaire and a verbal interview with a nurse. To facilitate reproducibility, the full details of the questionnaires and interview protocols can be accessed through the official UK Biobank documentation provided below:

**1. Touchscreen Questionnaire:**

This document details the questions presented to participants on the touchscreen device regarding their health, lifestyle, and demographics.

- **URL:** https://biobank.ctsu.ox.ac.uk/crystal/ukb/docs/TouchscreenQuestionsMainFinal.pdf

**2. Verbal Interview:**
This document outlines the procedures and questions asked during the verbal interview with the nurse, covering medical history and medications.

- **URL:** https://biobank.ctsu.ox.ac.uk/crystal/ukb/docs/Interview.pdf
